# *AKT1* and *BRAF* mutations in pediatric aggressive fibromatosis

**DOI:** 10.1002/cam4.669

**Published:** 2016-04-08

**Authors:** Cristina Meazza, Antonino Belfiore, Adele Busico, Giulio Settanni, Nicholas Paielli, Luca Cesana, Andrea Ferrari, Stefano Chiaravalli, Maura Massimino, Alessandro Gronchi, Chiara Colombo, Silvana Pilotti, Federica Perrone

**Affiliations:** ^1^ Pediatric Oncology UnitFondazione IRCCS Istituto Nazionale dei TumoriMilanItaly; ^2^ Laboratory of Experimental Molecular PathologyDepartment of PathologyFondazione IRCCS Istituto Nazionale dei TumoriMilanItaly; ^3^ Department of SurgeryFondazione IRCCS Istituto Nazionale dei TumoriMilanItaly

**Keywords:** *AKT1*, *BRAF*, *CTNNB1, TP53*, pediatric aggressive fibromatosis

## Abstract

Aside from the *CTNNB1* and adenomatous polyposis coli (APC) mutations, the genetic profile of pediatric aggressive fibromatosis (AF) has remained poorly characterized. The aim of this study was to shed more light on the mutational spectrum of pediatric AF, comparing it with its adult counterpart, with a view to identifying biomarkers for use as prognostic factors or new potential therapeutic targets. *CTNNB1*,*APC*,*AKT1*,*BRAF TP53*, and *RET* Sanger sequencing and next‐generation sequencing (NGS) with the 50‐gene Ion AmpliSeq Cancer Hotspot Panel v2 were performed on formalin‐fixed samples from 28 pediatric and 33 adult AFs. The prognostic value of *CTNNB1*,*AKT1*, and *BRAF* mutations in pediatric AF patients was investigated. Recurrence‐free survival (RFS) curves were estimated with the Kaplan–Meier method and statistical comparisons were drawn using the log‐rank test. In addition to the *CTNNB1* mutation (64%), pediatric AF showed *AKT1* (31%), *BRAF* (19%), and *TP53* (9%) mutations, whereas only the *CTNNB1* mutation was found in adult AF. The polymorphism Q472H *VEGFR* was identified in both pediatric (56%) and adult (40%) AF. Our results indicate that the mutational spectrum of pediatric AF is more complex than that of adult AF, with multiple gene mutations involving not only *CTNNB1* but also *AKT1* and *BRAF*. This intriguing finding may have clinical implications and warrants further investigations.

## Introduction

Aggressive fibromatosis (AF), also known as desmoid‐type fibromatosis, is a rare, locally aggressive nonmetastasizing fibroblastic proliferation with an incidence of 0.2–0.4 per 100,000 cases/year. It mainly affects young to middle‐aged adults, but children are also sometimes involved 1. Its growth may be fairly slow, lasting over several years and it can appear nearly anywhere in the body 2.

The pathogenesis of AF is not completely understood, although many factors can be implicated in its inception and progression, such as a genetic predisposition (familial adenomatous polyposis [FAP] and Gardner syndrome) 3.

The biological and clinical patterns of AF in children are generally considered the same as in adults and treatment recommendations are usually similar. Nowadays, a stepwise approach is generally adopted, involving “wait‐and‐see” and medical options, and local treatments that include nonmutilating surgery in selected cases 4, 5, 6, 7, 8, 9, 10.

As in adults, AF in children may occur sporadically or in association with FAP; in both settings, an activation of the Wnt/beta‐catenin signaling has been documented 11. Consistently, more than 60% of sporadic cases of pediatric AF show nuclear beta‐catenin expression and mutations of its gene *CTNNB1*, whereas syndromic pediatric AF exhibit adenomatous polyposis coli (*APC)* germline mutations 12.

The *CTNNB1* somatic mutations seen in sporadic pediatric AF are clustered in two codons and are represented exclusively by three different types of single‐nucleotide substitution leading to the amino acid changes T41A, S45F, and S45P. Intriguingly, Bo et al. 12. found that the S45F mutation occurred mainly in recurrent pediatric cases, whereas the T41A mutation was most frequently seen in primary‐onset cases, suggesting that S45F mutations may represent a risk factor for recurrence, as in surgically treated adult AF 13.

Aside from the *CTNNB1* and *APC* mutations, the genetic profile of pediatric AF has been poorly characterized. A few molecular analyses have been performed on adult AF using comparative genomic hybridization or high‐density single‐nucleotide polymorphism array, and shown loss of 5q (including the *APC* locus), 6q and 8p23, but such studies are completely lacking in pediatric AF 14, 15.

As in many other soft tissue sarcomas, it remains to be seen whether AF has the same biological background and the same clinical behavior when it occurs in children as opposed to adults.

The aim of this study was to gain some insight on the mutational spectrum of pediatric AF, comparing it with its adult counterpart, in an effort to identify potential biomarkers that might be used as prognostic factors or therapeutic targets.

## Material and Methods

### Patients and samples

Twenty‐eight primary pediatric AFs treated at the IRCCS Istituto Nazionale dei Tumori in Milan between 1990 and 2011 were considered in this study. The characteristics of the patients and their disease are described in Table 1. Eleven patients underwent biopsy, 13 nonradical surgery, and four radical surgery. Among patients underwent biopsy, four received chemotherapy (low‐dose chemotherapy, i.e., methotrexate and vinblastine in two patients and methotrexate and vinorelbine in three cases) immediately after diagnosis, while five patients received chemotherapy (methotrexate and vinorelbine in four cases and vincristine + actinomycin D + cyclophosphamide in one case) after evidence of progression.

**Table 1:** Clinical, *CTNNB1*, and adenomatous polyposis coli Sanger sequencing data in pediatric sporadic aggressive fibromatosis

N.	Sex/age	Type	Site	*CTNNB1*	*APC MCR*
1	f/15	Sporadic	Proximal lower limb	T41A	nd
2	m/3	Sporadic	H&N	T41A	nd
3	f/12	Sporadic	Proximal lower limb	T41A	nd
4	f/18	Sporadic	Trunk + Abdomen	T41A	nd
5	m/2	Sporadic	H&N	T41A	nd
6	f/6	Sporadic	H&N	T41A	nd
7	f/0.2	Sporadic	H&N	T41A	nd
8	m/18	Sporadic	Trunk	T41A + S45F	nd
9	m/2	Sporadic	Trunk + Distal upper limb	S45F	nd
10	m/9	Sporadic	H&N	S45F	nd
11	m/18	Sporadic	Distal lower limb	S45F	nd
12	f/12	Sporadic	Proximal upper limb	S45F	nd
13	f/18	Sporadic	Abdominal wall	S45F	nd
14	m/18	Sporadic	Distal lower limb	S45P	nd
15	f/12	Sporadic	Distal lower limb	S45P	nd
16	m/4	Sporadic	Proximal lower limb	S45P	nd
17	f/18	Sporadic	Proximal lower limb	S45P	nd
18	f/18	Sporadic	Distal lower limb	S45P	nd
19	m/14	Gardner syndrome	Abdominal wall	wt	wt
20	f/15	Sporadic	Proximal lower limb	wt	wt
21	f/15	Gardner syndrome	Abdomen	wt	wt
22	f/13	Sporadic	Trunk	wt	E1544K
23	m/5	Sporadic	Distal upper limb	wt	wt
24	m/7	Sporadic	Trunk	wt	wt
25	f/9	Sporadic	Distal upper limb	wt	wt
26	f/14	Sporadic	Distal lower limb	wt	na
27	f/14	Sporadic	Proximal lower limb	wt	na
28	3 months	Sporadic	H&N	wt	wt

f, female; m, male; wt, wild type; nd, not done; na, not assessable; H&N, head and neck; MCR, mutated cluster region.

The study sample was compared with a group of 33 surgically treated patients with sporadic adult AF, selected on the grounds of their known *CTNNB1* mutational status, whose characteristics are detailed in Table 2.

**Table 2:** Clinical, next‐generation sequencing (NGS), and Sanger sequencing data in adult aggressive fibromatosis

N.	Sex/age (years)	Type	Site	*CTNNB1*Sanger	NGS		
1	f/47	Sporadic	Abdominal wall	T41A	*CTNNB1* T41A		
2	f/60	Sporadic	Proximal upper limb	T41A	*CTNNB1* T41A		
3	m/27	Sporadic	Proximal lower limb	T41A	*CTNNB1* T41A		
4	f/32	Sporadic	Abdominal wall	S45F	*CTNNB1* S45F		
5	m/46	Sporadic	Trunk	S45F	*CTNNB1* S45F		
6	f/36	Sporadic	Abdominal wall	S45P	*CTNNB1* S45P		
7	f/35	Sporadic	Chest	wt	No mutation		
8	f/60	Sporadic	Peritoneum	wt	No mutation		
9	f/35	Sporadic	Abdominal wall	wt	No mutation		
10	f/65	Sporadic	Proximal lower limb	wt	*CTNNB1* T41A		
					Sanger sequencing		
					AKT1	BRAF	TP53
11	m	Sporadic	Chest wall	T41A	wt	wt	wt
12	f/32	Sporadic	Shoulder	T41A	wt	wt	wt
13	f/43	Sporadic	Abdomen	T41A	wt	wt	wt
14	m/34	Sporadic	Abdomen	T41A	wt	wt	wt
15	f/24	Sporadic	Abdominal wall	T41A	wt	wt	wt
16	f/31	Sporadic	Abdominal wall	T41A	wt	wt	wt
17	m/31	Sporadic	Trunk	T41A	wt	wt	wt
18	f/49	Sporadic	Mesentery	T41A	wt	wt	wt
19	f/28	Sporadic	Abdomen	T41A	wt	wt	wt
20	f/31	Sporadic	Abdominal wall	S45F	wt	wt	wt
21	f/42	Sporadic	Proximal lower limb	S45F	wt	wt	wt
22	f/35	Sporadic	Abdominal wall	S45F	wt	wt	wt
23	m/50	Sporadic	Proximal lower limb	S45F	wt	wt	wt
24	f/41	Sporadic	Abdominal wall	S45F	wt	wt	wt
25	f/31	Sporadic	Abdominal wall	S45P	wt	wt	wt
26	f/37	Sporadic	Abdominal wall	S45P	wt	wt	wt
27	f/34	Sporadic	Abdominal wall	S45P	wt	wt	wt
28	m/32	Sporadic	Abdomen	wt	wt	wt	wt
29	m/61	Sporadic	Trunk	wt	wt	wt	wt
30	f/70	Sporadic	Chest wall	wt	wt	wt	wt
31	f/36	Sporadic	Proximal lower limb	wt	wt	wt	wt
32	f/39	Sporadic	Abdominal wall	wt	wt	wt	wt
33	m/36	Sporadic	Abdomen‐retroperitoneum	wt	wt	wt	wt
				70%	0	0	0

f, female; m, male; wt, wild type.

### DNA extraction

Genomic DNA was extracted from 5 *μ*m sections cut from formalin‐fixed and paraffin‐embedded (FFPE) tumor samples with the GeneRead DNA FFPE kit (Qiagen, Hilden, Germany), according to the manufacturer's instructions.

### Sanger sequencing

PCR was performed using specific primers for *CTNNB1* (exon 3), *BRAF* (exon 15), and *TP53* (exon 8), as described elsewhere 16. The primers used to amplify *AKT1* (exon 2), *RET* (exon 11), and *APC* (the mutation cluster region in exon 15 where most of the mutations segregate) are listed in Table 3.

**Table 3:** Primers used for Sanger sequencing

Gene	Primers
Adenomatous polyposis coli (APC) EXON 15 PART 1	Fw 5′‐TGAAGAGAAACGTCATGTGGA‐3′
	Rev 5′‐CTTTGCAAGTGGCAGCCTTT‐3′
APC EXON 15 PART 2	Fw 5′‐AAGTGGTCAGCCTCAAAAGG‐3′
	Rev 5′‐GTGACACTGCTGGAACTTCG‐3′
APC EXON 15 PART 3	Fw 5′‐GATCCTGTGAGCGAAGTTCC‐3′
	Rev 5′‐AACATGAGTGGGGTCTCCTG‐3′
APC EXON 15 PART 4	Fw 5′‐ACACCCAAAAGTCCACCTGA‐3′
	Rev 5′‐ACTTCTCGCTTGGTTTGAGC‐3′
APC EXON 15 PART 5	Fw 5′‐AGCTCAAACCAAGCGAGAAG‐3′
	Rev 5′‐TTTCCTGAACTGGAGGCATT‐3′
APC EXON 15 PART 6	Fw 5′‐GCCTCCAGTTCAGGAAAATG‐3′
	Rev 5′‐ACAGGCAGCTGACTTGGTTT‐3′
APC EXON 15 PART 7	Fw 5′‐AGCCCAGACTGCTTCAAAAT‐3′
	Rev 5′‐TGCCCCTCCTCTAACTCCTT‐3′
AKT1 EXON 2	Fw 5′‐CGAAGGTCTGACGGGTAGAG‐3′
	Rev 5′‐CGCCACAGAGAAGTTGTTGA‐3′
RET EXON 11	Fw 5′‐AGGGATAGGGCCTGGGCTTC‐3′
	Rev 5′‐GACCTGGTTCTCCATGGAGTC‐3′

The PCR products were submitted to direct sequencing using a 3500 DX Genetic Analyzer (Applied Biosystems, Foster City, CA) and then assessed with the ChromasPro software.

### Next‐generation sequencing (NGS)

The 50‐gene Ion AmpliSeq Cancer Hotspot Panel v2 (Life Technologies) with the Ion‐Torrent^™^ Personal Genome Machine platform (Life Technologies, Foster city, CA, USA) was used in all experiments. This panel is designed to amplify 207 amplicons covering about 2800 COSMIC mutations from 50 oncogenes and tumor suppressor genes commonly mutated in human cancers (ABL1, AKT1, anaplastic lymphoma kinase (ALK), APC, ATM, BRAF, CDH1, CDKN2A, CSF1R, CTNNB1, EGFR, ERBB2, ERBB4, EZH2, FBXW7, FGFR1, FGFR2, FGFR3, FLT3, GNA11, GNAS, GNAQ, HNF1A, HRAS, IDH1, IDH2, JAK2, JAK3, KDR/VEGFR2, KIT, KRAS, MET, MLH1, MPL, NOTCH1, NPM1, NRAS, PDGFRA, PIK3CA, PTEN, PTPN11, RB1, RET, SMAD4, SMARCB1, SMO, SRC, STK11, TP53, VHL).

The Ion AmpliSeq Library Kit 2.0 (Life Technologies) was used to amplify 40 ng of DNA according to the manufacturer's instructions (MAN0006735 rev 5.0). The amplicons were partially digested with FuPa Reagent (Life Technologies), ligated to P1 and barcode adapters using DNA ligase. Barcoded libraries were purified using AMPure Beads XP (Beckman Coulter, Brea, CA, USA) and PCR‐amplified for a total of five cycles. After a second round of purification with AMPure Beads, the amplified libraries were measured for size and tested for quality using the Agilent Bio Analyzer DNA High Sensitivity kit (Agilent Technologies, Santa Clara, CA, USA), and quantified using the Qubit dsDNA HS kit (Invitrogen, Life Technologies, Carlsbad, CA, USA). Emulsion PCR and sample enrichment were completed using the IonOne Touch 2 instrument, the emulsion PCR master‐mix, the Ion sphere particles and Dynabeads MyOne Streptavidin C1 beads (Life Technologies), according to the manufacturer's instructions (Life Technologies). Sequencing was done on Ion Torrent PGM using Ion 316 Chips and the Ion‐PGM 200 sequencing kit (Life Technologies), according to the manufacturer's instructions.

PGM sequencing data were initially processed using the Ion Torrent platform‐specific Torrent Suite software to generate sequence readouts, align them on the reference genome Hg19, trim the adapter sequences, filter, and discard any poor signal‐profile readouts. Variant calling from the sequencing data was done with the Variant Caller plug‐in. The filtered variants were examined visually using the Integrative Genomic Viewer tool to check their quality level and confirm the variant's presence on both the “+” and the “−” strand. The resulting variants were then recorded using the Ensemble Variant Effect Predictor pipeline, COSMIC database, dbSNP database, and MyCancerGenome database (http://www.mycancergenome.org/). Information on the distribution of mutated genes in lung cancer was obtained from online catalogs of somatic mutations, such as Cosmic (http://cancer.sanger.ac.uk/cancergenome/projects/cosmic/), ClinVar of the National Center for Biotechnology Information (NCBI) (http://www.ncbi.nlm.nih.gov/clinvar/), the NCBI's Entrez Gene database (http://www.ncbi.nlm.nih.gov/entrez/query.fcgi?CMD=search&DB=gene), and the GeneCards of the Weizmann Institute of Science, Israel (http://www.genecards.org/).

### Statistical analysis

The prognostic analyses focused on the effects of the *CTNNB1*,* AKT1*, and *BRAF* mutations in pediatric AF patients. The study endpoint was recurrence‐free survival (RFS), and the time to the event was calculated from the date of surgery to the date of relapse or death, or it was censored as at the date of the latest uneventful follow‐up. RFS curves were estimated using the Kaplan–Meier method and were compared statistically using the log‐rank test.

## Results

### Pediatric AF mutation analysis

#### *β*‐catenin and *APC* Sanger sequencing

Twenty‐eight patients (M/F: 18/10) with a median age of 12 years (range 2 months–18 years) were assessed. Two patients with tumors arising in the abdominal wall had Gardner syndrome. The whole blood sample of only one of these two syndromic patients was subjected few years ago to the mutational analysis of the whole APC gene that revealed a germline mutation, as expected. This analysis was not performed for the second patient during its follow‐up.

The 28 FFPE samples of pediatric AF were analyzed for *CTNNB1* mutations by Sanger sequencing, which revealed this mutation in 18 (64%) cases (Table 1). The mutations found were as follows: ACC>GCC T41A (28%), TCT>TTT S45F (21%), and TCT>CCT S45P (18%) cases. Curiously, one case had both T41A and S45F mutations.

Since *CTNNB1* and *APC* mutations are generally mutually exclusive in AF, as in other tumors, nine of the 10 remaining cases carrying a *CTNNB1* wild type (wt) successfully underwent sequencing of APC. Since we analyzed FFPE samples, we sequenced exclusively the small part of the *APC* gene corresponding to the Mutated Cluster Region (MCR) and we found the missense mutation GAA>AAA E1544K in one tumor (10% of cases) (Table 1). The MCR of two patients with Gardner syndrome showed no mutation. Given that no peripheral blood lymphocytes were available for this retrospective study, it was impossible the sequencing of the whole *APC* to assess the occurrence of germline mutations in the two cases with Gardner syndrome, or to check for the absence of the E1544K *APC* mutation in normal tissue and thus exclude Gardner syndrome or FAP. The extended follow‐up nonetheless makes us confident of the clinical diagnosis of Gardner syndrome or sporadic AF.

#### NGS

To better characterize the genotype of pediatric AF, NGS was performed in 16 sporadic cases (six with T41A, two with S45F, four with S45P, one with T41A+S45F, and three with C*TNNB1* wt) using a panel of 50 oncogenes and tumor suppressor genes.

**Figure 1:**
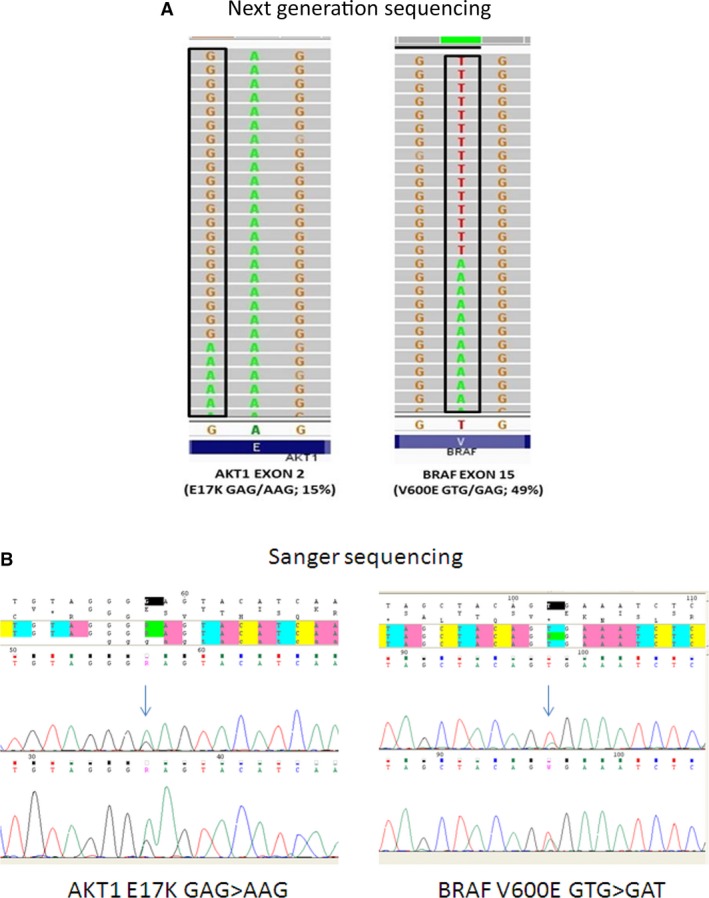
Mutational analysis in pediatric aggressive fibromatosis (AF). Next‐generation sequencing (NGS) (A) and Sanger sequencing (B) revealed *AKT1* E17K and *BRAF* V600E mutations.

In addition to confirming the findings for *CTNNB1* and *APC* obtained by Sanger sequencing, NGS revealed further mutations in five cases (31%), including: *AKT1* E17K (25%); *BRAF* V600E (12%) (Fig. 1A); *TP53* R273H (6%); and *RET* V648I (6%) (Table 4).

**Table 4:** Next‐generation sequencing in pediatric aggressive fibromatosis

	Mutation					Polymorphism				
N.	*CTNNB1*	AKT1	BRAF	TP53	*RET*	*VEGFR2*	*PI3KCA*	*TP53*	*FGFR3*	*KIT*
1	T41A	E17K	V600E	R273H			I391M			
2	T41A					Q472H				
3	T41A					Q472H				
4	T41A					Q472H	I391M			
5	T41A							P72R		
6	T41A		V600E							
8	T41A + S45F					Q472H		P72R		
9	S45F				V648I					M541L
11	S45F					Q472H		P72R		
14	S45P	E17K				Q472H				
15	S45P					Q472H				
17	S45P	E17K					I391M		F386L	
18	S45P								F386L	
20	wt									
23	wt	E17K				Q472H	I391M			
27	wt					Q472H	I391M			M541L
		25%	12%	6%	6%	56%	31%	19%	12%	12%

wt, wild type; Q472H, variant able to increase VEGFR2 phosphorylation after VEGFA stimulation in *vitro [* 25 *];* M541L, KIT variant with controversial impact on fibromatosis response to imatinib 17, 18, 19; P72R, p53 variant with high apoptotic potential and cytoplasmic localization able to influence the cytosolic functions of the p53 protein 29.

We also found some known polymorphisms such as the Q472H VEGFR2 (56%), I391M PIK3CA (31%), P72R TP53 (19%), F386L FGFR3 (12%), and M541L c‐KIT (12%) variants (Table 4). In particular, the M541L variant of c‐KIT has already been described in adult AF, where an association with AF tumorigenesis is unlikely, and its impact on response to imatinib remains controversial 17, 18, 19.

#### *AKT1*,* BRAF,* TP53, and *RET* Sanger sequencing

All the mutations found by NGS were confirmed by Sanger resequencing. Given the sizable proportion of *AKT1*,* BRAF*, and *TP53* mutations observed using NGS, these three genes were also sequenced directly in another 10 cases of pediatric AF. We were unable to analyze two cases because of the excessively small amount of tumor material available. *AKT1* and *BRAF* mutations were found in 40% and 30% cases, respectively (Fig. 1B). *TP53* analysis was successfully completed in only five cases, one of which (20%) carried the R273H mutation.

When the NGS and Sanger sequencing data were combined, after *CTNNB1, AKT1* emerged as the most frequently mutated gene (31%) (Table 5). The missense mutation E17K was seen in 2/7 T41A (28%), 2/4 S45P (50%), and 4/9 wt cases (44%), but in none of the S45F mutant AF. The *BRAF* V600E activating mutation occurred in five cases (19%), that is in 3/7 T41A (43%) and 2/9 wt AF (22%), whereas the *TP53* R273H mutation was observed in two (9%) cases. The *APC* mutation occurred in only one case (4%).

**Table 5:** Combined Sanger and next‐generation sequencing results in pediatric aggressive fibromatosis

	Mutation				
N.	*CTNNB1*	*APC MCR*	AKT1	BRAF	TP53
1	T41A	wt	E17K^a^	V600E^a^	R273H^d, nf^
2	T41A	wt	wt	wt	wt
3	T41A	wt	wt	wt	wt
4	T41A	wt	wt	wt	wt
5	T41A	wt	wt	wt	wt
6	T41A	wt	wt	V600E^a^	wt
7	T41A	ND	E17K^a^	V600E^a^	na
8	T41A + S45F	wt	wt	wt	wt
9	S45F	wt	wt	wt	wt
10	S45F	ND	wt	wt	na
11	S45F	wt	wt	wt	wt
12	S45F	wt	wt	wt	wt
13	S45F	ND	wt	wt	wt
14	S45P	wt	E17K^a^	wt	wt
15	S45P	wt	wt	wt	wt
16	S45P	ND	na	na	na
17	S45P	wt	E17K^a^	wt	wt
18	S45P	wt	wt	wt	wt
19	wt	wt	wt	V600E^a^	R273H^d, nf^
20	wt	wt	wt	wt	wt
21	wt	wt	wt	wt	na
22	wt	E1544K	E17K^a^	wt	na
23	wt	wt	E17K^a^	wt	wt
24	wt	wt	E17K^a^	V600E^a^	wt
25	wt	wt	na	na	na
26	wt	na	wt	wt	wt
27	wt	wt	wt	wt	wt
28	wt	wt	E17K^a^	wt	na
	64%	4%	31%	19%	9%

na, not assessable; ND, not done; wt, wild type; *APC,* adenomatous polyposis coli; MCR, mutated cluster region; a, activating mutation; d, disruptive mutation 30; nf, nonfunctional mutation 31

Considering all the genes analyzed, there were 14 cases with one mutation, six with two, one with three, and one with four mutations, while five cases revealed none.

### Adult AF mutational analysis

#### NGS

We analyzed 10 adult sporadic AFs selected on the grounds of their previously established *CTNNB1* mutational status (three T41A, two S45F, one S45P, and four wt). No mutations other than *CTNNB1* came to light (Table 2). Interestingly, NGS revealed the T41A mutation in a case classified as wt on Sanger sequencing (case number 10). The only polymorphism observed was Q472H VEGFR2 (40%).

#### *AKT1*,* BRAF*, and *TP53* Sanger sequencing

Twenty‐three additional adult cases of sporadic AF (nine T41A; five S45F; three S45P, six wt) were submitted to Sanger sequencing of the three additional genes found mutated in pediatric AF (Table 2), and they all proved to be wt for *AKT1*,* BRAF*, and *TP53*.

Pooling the NGS and Sanger sequencing data, 36 sporadic adult AFs were analyzed successfully and no mutations other than *CTNNB1* were found.

### Recurrence‐free survival in pediatric AF

The prognostic analyses focused on the effects of *β*‐catenin, *AKT1*, and *BRAF* mutations in pediatric patients. With a median follow‐up of 93 months (range between 21–246 months), the estimated 3‐year and 5‐year RFS rates were 45.5%, for the series as a whole.

**Figure 2:**
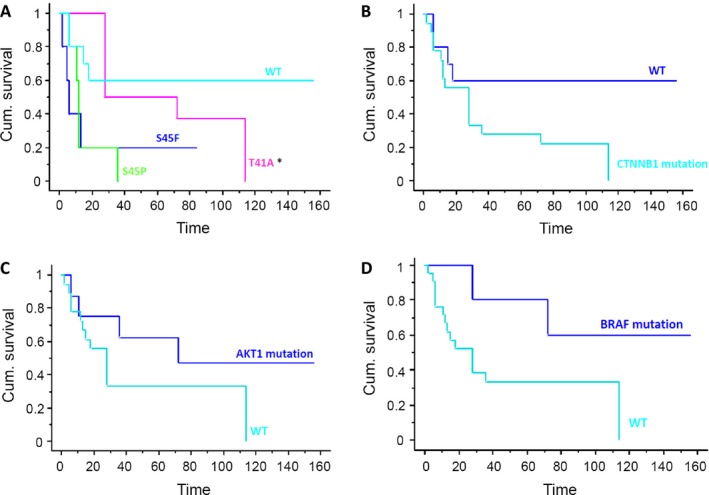
Recurrence‐free survival (RFS) curves in pediatric aggressive fibromatosis (AF). RFS is illustrated in patients by specific *CTNNB1* mutations (A) *CTNNB1* mutation versus wild type (B) *AKT1* mutation (C) *BRAF* mutation (D).

For the patients with *CTNNB1* mutations, the estimated 3‐year and 5‐year RFS rates were: 20% at both time points for patients with the 45F mutation; 43% for patients with the 41A mutation; and 20% and 0% for patients with the 45P mutation. Patients who were wt for *CTNNB1* mutations had a 3‐year and 5‐year RFS rate of 60% at both time points. (Fig. 2A). When wt and all *CTNNB1* mutated patients were compared, the 3‐ and 5‐year RFS rates were 60% and 28%, respectively (*P *= 0.078; Fig. 2B).

Eight patients had a *AKT1* mutation: the estimated 3‐year and 5‐year RFS rates for these patients were both 62.5%, as opposed to 30% for the *AKT1* wt patients (*P *= 0.02469) (Fig. 2C).

Five patients had a *BRAF* mutation: the estimated 3‐year and 5‐year RFS rates for these patients were 80% and 60%, as opposed to 33% for the *BRAF* wt patients (*P *= 0.1058) (Fig. 2D).

**Figure 3:**
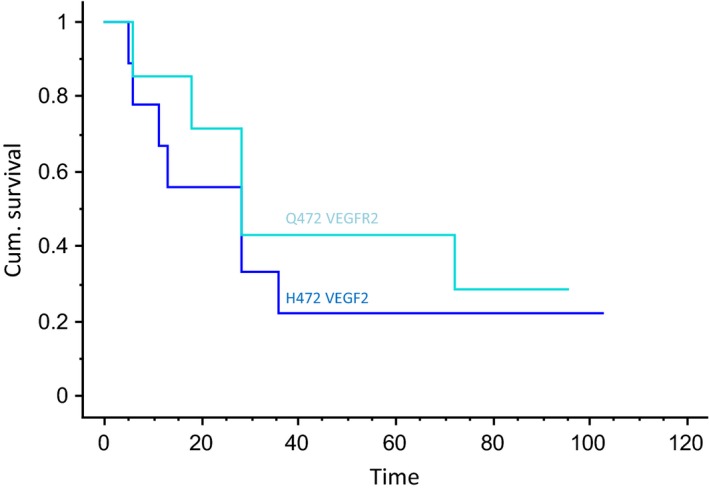
Recurrence‐free survival (RFS) curves in pediatric aggressive fibromatosis (AF) according to Q472H VEGFR2 polymorphism.

Nine patients had Q472H *VEGFR2* polymorphism: the estimated 3‐year and 5‐year RFS rates for H472 patients were both 22%, as opposed to 43% for Q472 patients (*P *= 0.38) (Fig. 3).

We reported that five patients did not have any aforementioned mutations (cases #20, 21, 25, 26, and 27): the estimated 3‐year and 5‐year RFS for this group was 40%, as opposed to 36% for mutated patients (*P *= 0.9).

## Discussion

In our study, we obtained sequencing data on 50 cancer‐related genes in cases of pediatric AF and compared them with findings in the adult counterpart of AF, identifying significant differences mainly involving *AKT1* and *BRAF* mutations in pediatric AF.

This retrospective series of 28 pediatric AFs was characterized for Wnt/beta‐catenin alterations. As in the adult counterpart 13, *CTNNB1* genotyping revealed a particularly high rate (64%) of mutations in the two codons T41 and S45. In detail, the distribution of the types of mutation that we identified suggests a similar frequency between T41A (28%) and S45F (21%), unlike two previous studies reporting that T41A was the most common type of mutation seen in pediatric AF 11, 12, as in adult AF 13. We nonetheless confirmed that S45P (14%) was the least common mutation, in line with the literature 11. Based on our previous experience with 179 cases of completely resected, sporadic primary AF, judging from which tumors with the S45F mutation have a higher tendency for local recurrence 13, we explored the possible prognostic role of specific mutations in pediatric AF. We found no statistically significant difference in terms of RFS for wt cases, though there was evidence of a trend (Fig. 2B). Unfortunately, our results failed to demonstrate that S45F or any of the *CTNBB1* mutations could be useful as a molecular indicator of a higher likelihood of recurrence (probably because the number of patients considered here was not enough to reveal differences relating to the different types of mutation). Similar data were obtained by Wang 11, whereas one study found the S45F mutation more frequent in recurrent pediatric AF than in primary tumors 12. This matter deserves further investigation to establish clearly whether these different types of mutation confer a different phenotype, and whether *CTNNB1* mutational status might therefore be used as prognostic tool.

To broaden our limited molecular understanding of AF and seek potential new biomarkers for targeted therapy, we better characterized the pediatric AF genotype by performing NGS and Sanger sequencing, and comparing the results with findings in adult AF. To the best of our knowledge, this is the first report of the E17K *AKT1* mutation (31%) occurring frequently in pediatric AF. This mutation was found in *CTNNB1* wt and T41A and S45P mutant pediatric cases, but not in patients with the S45F mutation.

AKT1 kinase is one of the most frequently activated survival pathways in cancer, and the well‐studied E17K mutation in the pleckstrin homology (PH) domain of AKT1 stimulates downstream signaling and transforms cells, causing AKT1 activation by means of a rapid conformational drift that in turn increases the localization of the protein on the plasma membrane 20, 21. The presence of *AKT1* mutation might have clinical implications based on the preclinical evidence that this mutant has been found resistant to AKT1/2 inhibitor, suggesting that pediatric AF patients probably would not benefit from this target treatment 20.

A small proportion of our *CTNNB1* mutant or wt pediatric AF cases (19%) harbored the common *BRAF* V600E substitution that destabilizes this kinase's inactive conformation, leading to its constitutive activation. This finding deserves further investigation in order to explore BRAF as a potential therapeutic target in pediatric AF due to the current availability of several BRAF inhibitors.

We found a higher RFS rate for the *AKT1* or *BRAF* mutated cases of no statistical significance.

The R273H *TP53* mutation was observed in 2 (9%) cases. It is worth noting that—both in sporadic and FAP‐related adult AF—previous studies investigated p53 protein expression only by immunohistochemistry, showing that nuclear p53 immunostaining was associated with a higher risk of tumor recurrence 22, 23.

Interestingly, no *AKT1*,* BRAF*, or TP53 mutations were found in the series of 33 adult AFs that we selected on the grounds of their *CTNNB1* status; this would suggest that such alterations are a distinctive feature of pediatric AF that should be validated on independent series.

One pediatric case (6%) also revealed the V648I mutation in the human *RET* oncogene, a rare substitution reported in medullary thyroid carcinoma, a setting in which it was judged to be “non‐transforming” in the light of elegant *in silico* and *in vitro* analyses, and this raised some doubts as to whether V648I might represent the driving force behind the tumor 24. Although in AF, we cannot say for sure whether the ability of V648I to induce a tumoral transformation is related to particular predisposing genetic conditions, this mutation seems to have a marginal role in pediatric AF.

In a sizable proportion of both pediatric (56%) and adult (40%) cases of AF, NGS also revealed the Q472H VEGFR2 polymorphism that was the only genetic variant capable of increasing protein phosphorylation after VEGFA stimulation *in vitro* 25. The effect of Q472H on VEGFR2 function may be due to a more efficient binding to the ligand. The role of VEGFR2 in carcinogenesis and tumor progression is related to its well‐known proangiogenic effects, and the PDGFR‐B expression that we observed by IHC in all pediatric AF cases may go in the same direction, along with COX expression (data not shown). These results are in line with our previous findings of PDGFRB expression/phosphorylation in frozen samples of adult AF, and of PDGFRA too (in some of them at least), probably mediated by their cognate ligands 26. Unfortunately, we were unable to test the activation of these two receptors in our pediatric AF series owing to a shortage of frozen material. We speculate that the PDGFR expression, along with the Q472H VEGFR2 variant and the mutation‐mediated BRAF activation, might have a proangiogenic role in pediatric AF, favoring a response to multitarget anti‐angiogenic agents found active in the adult counterpart 27, 28.

In conclusion, our results suggest that the mutational spectrum of pediatric AF is more complex than in adult AF, being rich in *AKT1* and *BRAF,* as well as *CTNNB1* gene mutations. These intriguing findings could have clinical implications and warrant further investigation on a new and larger cohort of patients. We hopefully expect that our study could represent the rationale for a future international collaboration between pediatric oncologists involved in a so rare and peculiar disease.

## Conflict of Interest

None declared.
